# A phase II study evaluating the efficacy of enzalutamide and the role of liquid biopsy for evaluation of ARv7 in mCRPC patients with measurable metastases including visceral disease (Excalibur study)

**DOI:** 10.1177/17588359231217958

**Published:** 2024-01-22

**Authors:** Pierangela Sepe, Giuseppe Procopio, Chiara Carlotta Pircher, Umberto Basso, Orazio Caffo, Vera Cappelletti, Melanie Claps, Ugo De Giorgi, Lucia Fratino, Valentina Guadalupi, Patrizia Miodini, Cinzia De Marco, Bruno Perrucci, Alessia Mennitto, Daniele Santini, Francesco Spina, Marco Stellato, Filippo de Braud, Elena Verzoni

**Affiliations:** Medical Oncology Unit, Fondazione IRCCS Istituto Nazionale dei Tumori di Milano, Via Giacomo Venezian 1, Milan 20133, Italy; Medical Oncology Unit, Fondazione IRCCS Istituto Nazionale dei Tumori di Milano, Milan, Italy; Programma Prostata, Fondazione IRCCS Istituto Nazionale dei Tumori di Milano, Milan, Italy; Medical Oncology Unit, Fondazione IRCCS Istituto Nazionale dei Tumori di Milano, Milan, Italy; Oncology Unit 1, Department of Medical Oncology, Istituto Oncologico Veneto IOV IRCCS, Padova, Italy; Department of Medical Oncology, Santa Chiara Hospital, Trento, Italy; Department of Advanced Diagnostics, Fondazione IRCCS Istituto Nazionale Tumori di Milano, Milan, Italy; Medical Oncology Unit, Fondazione IRCCS Istituto Nazionale dei Tumori di Milano, Milan, Italy; Department of Medical Oncology, IRCCS Istituto Romagnolo per lo Studio dei Tumori (IRST) Dino Amadori, Meldola, Italy; Department of Medical Oncology, Centro di Riferimento Oncologico di Aviano, IRCCS, Aviano, Italy; Medical Oncology Unit, Fondazione IRCCS Istituto Nazionale dei Tumori di Milano, Milan, Italy; Department of Advanced Diagnostics, Fondazione IRCCS Istituto Nazionale Tumori di Milano, Milan, Italy; Department of Advanced Diagnostics, Fondazione IRCCS Istituto Nazionale Tumori di Milano, Milan, Italy; Oncology Department, ASST Istituti Ospitalieri, Cremona, Italy; Department of Medical Oncology, University Hospital Maggiore della Carità, Novara, Italy; Medical Oncology, Department of Translational Medicine (DIMET), University of Eastern Piedmont (UPO), Novara, Italy; Oncologia Medica, Campus Bio-Medico University of Rome, Rome, Italy; University of Rome La Sapienza, Roma, Italy; Department of Hematology and Oncology, Niguarda Cancer Center, Ospedale Niguarda Ca’ Granda, Milan, Italy; Medical Oncology Unit, Fondazione IRCCS Istituto Nazionale dei Tumori di Milano, Milan, Italy; Medical Oncology Unit, Fondazione IRCCS Istituto Nazionale dei Tumori di Milano, Milan, Italy; Department of Oncology and Hemato-Oncology, University of Milan, Milan, Italy; Medical Oncology Unit, Fondazione IRCCS Istituto Nazionale dei Tumori di Milano, Milan, Italy

**Keywords:** androgen receptor signaling inhibitor, ARv7, CTC, enzalutamide, liquid biopsy, metastatic castration-resistant prostate cancer, overall survival, radiographic progression-free survival, visceral

## Abstract

**Background:**

Up to 30% of patients with metastatic castration-resistant prostate cancer (mCRPC) develop visceral metastases, which are associated with a poor prognosis.

**Objectives:**

Efficacy of enzalutamide in mCRPC patients with measurable metastases, including visceral and/or extra-regional lymph nodes.

**Methods:**

In this phase II multicenter study, patients with mCRPC and measurable metastases received enzalutamide as the first line. Primary endpoint: 3-month (mo) disease control rate (DCR) defined as the proportion of patients with complete (CR) or partial response (PR) or stable disease (SD) as per Response Evaluation Criteria in Solid Tumors 1.1. Secondary endpoint: safety. Exploratory endpoint: the association between ARv7 splicing variants in basal circulating tumor cell (CTC)-enriched blood samples and treatment response/resistance using the AdnaTest ProstateCancerSelect kit and the AdnaTest ProstateCancer Panel AR-V7.

**Results:**

From March 2017 to January 2021, 68 patients were enrolled. One patient never started treatment. Median age: 72 years. A total of 52 patients (78%) received enzalutamide as a first line for mCRPC. The median follow-up was 32 months. At the 3-month assessment, 24 patients presented an SD, 1 patient achieved a CR, and 23 patients had a PR (3-mo-DCR of 72%). Discontinuations due to adverse events (AEs), disease-related death, or disease progression occurred in 9%, 6%, and 48% of patients. All patients reported at least one grade (G) 1–2 AE: the most common were fatigue (49%) and hypertension (33%). Six G3 AEs were reported: two hypertension, one seizure, one fatigue, one diarrhea, and one headache. Basal detection of ARv7 was significantly associated with poor treatment response (p = 0.034) and a nonsignificant association (p = 0.15) was observed between ARv7 detection and response assessments. At month 3, ARv7 was detected in 57%, 25%, and 15% of patients undergoing progressive disease, SD, and PR, respectively.

**Conclusion:**

The study met its primary endpoint, showing the efficacy of enzalutamide in men with mCRPC and measurable metastatic lesions in visceral and/or lymph node sites.

**Trial registration:**

ClinicalTrials.gov Identifier: NCT03103724. First Posted: 6 April 2017. First patient enrollment: 19 April 2017.

## Introduction

Prostate cancer ranks as the second most prevalent malignancy among men, accounting for 7% of all newly diagnosed cancers in men worldwide.^1^ While patients with recurrent prostate cancer or *de novo* metastatic disease initially respond to androgen deprivation therapy, a significant proportion eventually develop castration-resistant prostate cancer (CRPC) within 1–3 years.^2^ Metastatic CRPC (mCRPC), an advanced stage of the disease, presents with a multifaceted clinical landscape, with approximately 10–30% of patients manifesting visceral metastases, notably in the lung and/or liver.^3^ The presence of visceral disease has traditionally been considered a negative prognostic factor in mCRPC.^4,5^ In this challenging scenario, hormonal therapies such as enzalutamide or abiraterone have shown promise by extending survival across various prostate cancer stages, including mCRPC.^6–10^ However, a crucial gap in knowledge persists, as there is a scarcity of prospective trials designed specifically to address the efficacy and safety of these hormonal agents in visceral disease in the castration phase. The COU-AA-302 trial,^7^ which assessed abiraterone acetate in chemotherapy-naïve men with mCRPC, notably excluded patients with visceral disease, leaving a critical void in the understanding of this patient subgroup. On the contrary, *post hoc* analysis of the COU-AA-301^11^ trial comparing abiraterone acetate *versus* placebo in post-docetaxel mCRPC included 352 (29.5% of the study cohort) patients with visceral disease, which included liver, lung, or other soft tissue metastases at baseline, whether or not bone or nodal sites of disease were also present. Here, patients showed similar benefits with abiraterone acetate in men with or without visceral disease.^11^ Similarly, the AFFIRM trial,^12^ a post-docetaxel study of enzalutamide, showcased the independence of liver metastases as a predictor of overall survival (OS), highlighting the efficacy of treatment regardless of visceral disease presence. Furthermore, the PREVAIL study,^13^ which compared enzalutamide to placebo in chemotherapy naïve mCRPC, reported benefits for patients with visceral disease (12% of the study cohort), further affirming the potential of enzalutamide in this context. Concurrently, advancements in the treatment of metastatic hormone-sensitive prostate cancer (mHSPC) have revolutionized the treatment landscape, showing the remarkable efficacy of new hormonal agents like abiraterone, apalutamide, and enzalutamide, even in the presence of visceral disease.^14,15^ At the time when the Excalibur study was designed, it is important to highlight that only docetaxel was available for prescription as a treatment option for mHSPC. Taken together, the aforementioned data substantiate enzalutamide as a rational treatment option for men with mCRPC and visceral metastases.

Nevertheless, it remains a stark reality that approximately 20–40% of patients exhibit primary resistance to these treatments,^7–9,16^ while others eventually develop secondary resistance,^17^ underscoring the urgency of understanding the mechanisms underlying treatment response and resistance.

In this context, liquid biopsy has emerged as a valuable tool for assessing prognostic biomarkers and monitoring treatment responses in various malignancies, especially in advanced stages where obtaining tissue samples is often challenging.^18–21^ Over the past decade, a multitude of androgen receptor (AR) splice variants, including ARv7, have been identified as a potential contributor to the development of resistance to androgen deprivation and AR-signaling inhibitors.^17^ ARv7, in particular, a constitutively active splicing variant of AR lacking the ligand-binding domain, holds significant promise as a clinically valid biomarker.^22^ The detection of ARv7 in liquid biopsy samples has exhibited an association with treatment failure, suggesting its potential for patient stratification in clinical trials.^23,24^ Despite many investigations into ARv7 in the context of mCRPC, the predictive relevance of ARv7 has yet to be specifically addressed in the unique context of visceral metastases. Therefore, the Excalibur study was designed to bridge this gap by prospectively assessing the efficacy of the second-generation AR inhibitor enzalutamide as both first- or second-line treatment for mCRPC patients with at least one measurable site, including visceral metastasis and/or extra-regional lymph nodes. Simultaneously, we aimed to explore the association between the detection of liquid biopsy-derived ARv7 and treatment response/resistance.

## Materials and methods

### Study participants

Eligible patients had histological confirmation of adenocarcinoma of the prostate and at least one metastatic measurable lesion as defined by Response Evaluation Criteria in Solid Tumors (RECIST) version 1.1, including those in the lung, liver, or/and extra-regional lymph nodes. Patients must have shown prostate-specific antigen (PSA) progression, radiographic progression, or both in bone or visceral sites, in accordance with Prostate Cancer Clinical Trials Working Group 3 criteria, despite receiving hormone-releasing hormone (LH-RH) analog therapy or undergoing orchiectomy, with a serum testosterone level of 1.73 nmol/L (50 ng/dL) or lower. Previous therapy with docetaxel for hormone-sensitive or castration-resistant phases was permitted if the last cycle was received 3 weeks before the start of the study treatment. Previous radiotherapy to the prostate and/or bone was acceptable if it was discontinued at least 3 weeks before starting the study treatment. Hormonal treatment containing bicalutamide had to be discontinued at least 2 weeks before starting the study therapy. Further inclusion criteria were Eastern Cooperative Oncology Group (ECOG) performance status (PS) of 2 or lower. Patients with a history of a seizure or a condition that could predispose to seizure were excluded, although patients taking medications associated with lowering the seizure threshold were eligible.

The study was approved by the independent review board at each participating site and was conducted in accordance with the International Council for Harmonization Good Clinical Practice Guidelines and the Declaration of Helsinki. Institutional review boards or independent ethics committees approved the study. All patients provided written informed consent before study procedures.

### Study design and treatment

The Excalibur study (Institutional review board approved with the number INT 178/15) was an open-label, single-arm, prospective phase II study carried out between March 2017 and January 2021. It involved six different Italian Oncology Units under the coordination of Fondazione IRCCS Istituto Nazionale Tumori in Milan, Italy.

All patients received oral enzalutamide orally at the dose of 160 mg once daily, with or without food. Each cycle of treatment lasted 28 days, and patients could continue treatment until experiencing unacceptable side effects, confirming radiographic progression, or withdrawing their consent. Treatment discontinuation solely due to a rise in PSA levels was discouraged.

The primary objective of the trial was to assess clinical benefit, as determined by the 3-month disease control rate (3-mo-DCR) provided by enzalutamide as first- or second-line treatment for patients with mCRPC who had at least one measurable metastatic lesion as defined by RECIST 1.1, including sites in the lung, liver, and/or extra-regional lymph nodes metastases.

### Study endpoints

The primary endpoint was the 3-mo-DCR defined as the sum of complete response (CR), partial response (PR), and stable disease (SD) according to RECIST 1.1 after 3 months of study treatment. The secondary endpoint was the safety. The exploratory objective was to investigate the association between ARv7 splicing variants [in circulating tumor cell (CTC) samples] and treatment response/resistance. Data on patient-reported outcomes were collected but are not reported in this document.

### Study assessments

After providing written informed consent, patients underwent screening procedures to determine their eligibility. These procedures encompassed a thorough review of the patient’s medical history, a comprehensive physical examination, measurements of vital signs, a review of concomitant therapies, and clinical blood laboratory tests including PSA concentration. In addition, an electrocardiogram was conducted, and the cardiac ejection fraction was assessed by echocardiogram. Radiological assessments included a bone scan and a computed tomography or magnetic resonance imaging (MRI) of the chest, abdomen, and pelvis. Following enrollment, scheduled visits occurred according to clinical practice every 4 weeks, unless clinically indicated. On the first day of each cycle (every 4 weeks), during the treatment period (±7 days) several assessments were performed, including physical examination vital signs monitoring, ECOG-PS evaluation, review of concomitant medications, and laboratory evaluations in accordance with clinical practice.

Efficacy assessments included monitoring the PSA concentrations and radiological assessments performed at the screening and, subsequently every 3 months from the date of study treatment start, as per clinical practice. All additional suspected sites of disease should be imaged. For safety assessments, all adverse events (AEs) and serious AEs were monitored and recorded with the use of the Common Terminology Criteria for Adverse Events, version 4.03. The AEs summarized and discussed in this report are those that emerged during the treatment period, up to 30 days after the last dose of the trial drug.

### ARv7 detection

Peripheral whole blood samples were collected using K_3_EDTA BD Vacutainer tubes or BD Vacutainer ACDA tubes (Becton Dickinson GmBH, Venlo, The Netherlands) for patients recruited at INT and in the peripheral centers, respectively. Blood collection was performed at the start of treatment and, when possible, during treatment and at progression. CTC detection and ARv7 evaluation have been previously described.^23,24^ Briefly, CTCs were enriched from 5 mL of whole blood using the AdnaTest ProstateCancerSelect kit (AdnaGen, AG, Langenhagen, Germany) exploiting tumor-specific and epithelial markers, whereas the AdnaTest ProstateCancerDetect kit was used for detection of CTCs. Samples were classified as CTC status positive (CTC^+ve^) when at least one of the tumor-specific markers (*PSMA, PSA*) was above the threshold defined by the manufacturer (0.10 ng/µL). The expression of *AR* and *ARV7* was evaluated on the same cDNA samples prepared for CTC status determination described above using the AR-V7 RTPCR kit (AR-V7 assay RT-PCR, Bird, Monteriggioni, Italy) according to the manufacturer’s instructions or using the AdnaTest ProstateCancerPanel AR-V7 (Qiagen). Values were reported as positive or negative using a threshold of ⩾10 copies/mL for the Bird kit and referring to the instructions of the AdnaTest ProstateCancerSelect AR-V7 Handbook for the Qiagen kit. CTC, AR, and ARv7 determinations were run without the knowledge of clinical data.

### Statistical analysis

Continuous variables were presented as mean values ± standard deviations or median values (interquartile ranges), and categorical variables were reported as numbers and percentages. Percentages were expressed in relation to the total population unless otherwise specified. Median follow-up was calculated by the reverse Kaplan–Meier approach. Survival curves for progression-free survival (PFS) and OS were estimated using the Kaplan–Meier method and compared by the log-rank test. The primary endpoint was 3-mo-DCR. The A'Hern’s Single Stage Phase II design has been used to test the null hypothesis that the true 3-mo-DCR rate is ⩽35%, as opposed to the alternative hypothesis of ⩾50%. Assuming a type I error rate of 5% and power of 80%, 31 out of 68 patients will be required to be in disease control at 3 months to consider the agent worthy of further investigation.

Safety analyses were performed for patients who received at least one dose of treatment. The safety variables of the treatment have been summarized according to the grade of NCI-CTCA v. 5.0.

Statistical calculations were performed with R software, version 3.4.1 (R Foundation for Statistical Computing).

## Results

### Patients

**Figure 1.**
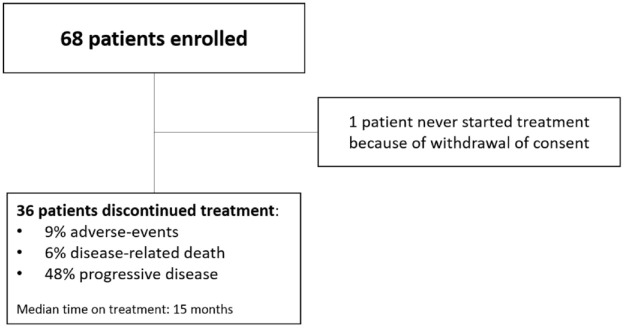
Consort diagram.

Between March 2017 and January 2021, the study enrolled 68 patients. One patient never started treatment due to consent withdrawal (Figure 1). The median age of participants was 72 years (range, 52–88 years). All patients had at least one measurable metastatic lesion in the lungs and/or liver and/or extra-regional lymph nodes, as defined by RECIST 1.1. Specifically, 36 (54%) patients had only extra-regional nodal metastases. Among the remaining 31 patients, 9 had visceral metastases (liver, *n* = 1; lung, *n* = 7; liver and lung *n* = 1). In all, 22 patients had visceral metastases (liver, *n* = 2; lung, *n* = 17; both liver and lung *n* = 3) along with coexistent extra-regional nodal metastases. Bone metastases were present in 37 patients (55%).

The majority of patients (98%) had ECOG-PS of 0 or 1. In total, 15 patients (22%) had received previous docetaxel treatment for mCRPC, making enzalutamide their second-line therapy. The remaining 52 patients (78%) received enzalutamide as their first-line therapy for mCRPC. None of the patients included in the study had received previous treatment with new hormonal agents.

All patients were on androgen-deprivation therapy with LH–RH analogs or antagonists. Over two-thirds of the patients had at least one comorbidity at the start of treatment with 52% having stable and well-compensated cardiovascular disorders (49% with hypertension) and 20% having metabolic disorders (12% with type 2 diabetes and 8% with hypercholesterolemia).

Baseline demographic and disease characteristics are summarized in Table 1.

**Table 1.** Baseline characteristics of patients.

Patients – *n*	67
Age	
Median (range) – years	72 (52–88)
Eastern Cooperative Oncology Group performance status – *n* (%)	
0–1	66 (99)
2	1 (1)
Previous docetaxel – *n* (%)	
Yes	15 (22)
No	52 (78)
Disease location – *n* (%)	
Only extra-regional lymph nodes	36 (54)
Visceral non-nodal	9 (13)
Liver	1 (1)
Lungs	7 (10)
Lungs + liver	1 (1)
Visceral non-nodal + extra regional nodal:	22 (33)
Lung + liver + lymph nodes	3 (5)
Liver + lymph nodes	2 (3)
Lung + lymph nodes	17 (25)
Bone	37 (55)
Number of metastatic sites – *n* (%)	
1	26 (39)
2	22 (33)
>2	19 (28)
TNM at initial diagnosis – *n* (%)	
M0 (progressed after local treatment)	57 (85)
M1 (*de novo*)	10 (15)

The median duration of treatment was 15 months [95% confidence interval (CI): 12–21]. In all, 33 patients received at least 12 months of treatment and as of the data-cutoff date in September 2022, 14 patients were continuing to receive treatment.

### Tumor response

After 3 months of treatment, 36% (24/67) of the patients had an objective response with CR and PRs observed in 1% (1/67) and 34% (23/67) of patients, respectively. In all, 24 patients (36%) presented an SD resulting in a 3-mo-DCR of 72% (48/67). Tumor responses are summarized in Table 2. Enzalutamide produced a DCR of 80% in patients with extra-regional nodal metastases only (29/36). In the remaining 31 patients with lung and/or liver involvement, with or without extra-regional nodal metastases enzalutamide produced DCR in 19 of them (61%). Tumor responses stratified for metastatic sites are summarized in Table 3. Overall, after 3 months of treatment, reductions of at least 50% in PSA levels were observed in 64% (43/67) of the patients. This response was maintained at month 6 and month 12, with a median time to PSA response of 2 months.

**Table 2.** Summary of tumor response.

Best overall response at month 3, *n* (%)	
CR	1 (1)
PR	23 (34)
SD	24 (36)
PD	9 (13)
Unknown/not evaluable	10 (15)
3-mo-DCR (CR + PR + SD), *n* (%)	48 (72)
Time on treatment, median, months	15
rPFS, median time	17 (95% CI: 13–25).
mOS, median time	33 (95% CI: 30–NA).

3-mo-DCR, 3 months disease control rate; CR, complete response; mOS, median overall survival; PD, progressive disease; PR, partial response; rPFS, radiological progression-free survival; SD, stable disease.

**Table 3.** Summary of tumor response according to metastatic sites.

Disease location	*n* (%)	3-mo-DCR (CR + PR + SD), *n* (%)
Only extra-regional lymph nodes	36 (54)	29 (80)
Visceral	9 (13)	5 (55)
Liver	1 (1)	NA
Lung	7 (10)	5
Lung + liver	1 (1)	0
Visceral + extra-regional nodal	22 (33)	14 (42)
Lung + liver + lymph nodes	3 (5)	0
Liver + lymph nodes	2 (3)	2
Lung + lymph nodes	17 (25)	12

3-mo-DCR, 3 months disease control rate; CR, complete response; NA, not available; PR, partial response; SD, stable disease.

### Progression-free survival and OS

**Figure 2.**
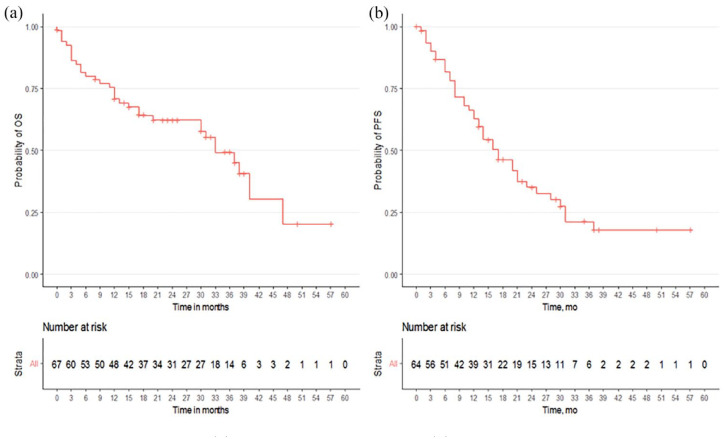
Kaplan–Meier curves: (a) progression-free survival and (b) overall survival.

With a median follow-up of 32 months, rPFS was 17 months (95% CI: 13–25) and the median OS was 33 months (95% CI: 30–NA). Survival outcomes are displayed in Figure 2 and Table 2. Subsequent antineoplastic treatments were received by 28% (19/67) of patients. The most common subsequent therapy was chemotherapy with docetaxel or cabazitaxel received by 47% and 16% of patients, respectively. The duration and efficacy of post-progression therapies were not ascertained. Discontinuations due to AEs, disease-related death, or disease progression occurred in 9% (6/67), 6% (4/67), and 48% (32/67) of patients, respectively (Figure 1). The most common site of progression was in the lymph nodes, followed by the bone.

Next, we examined the prognostic impact of metastatic disease sites (lungs and/or liver with or without coexistent extra-regional nodal metastases subset *versus* extra-regional nodal metastases only subset). The median OS with enzalutamide in the subset with lungs and/or liver and coexistent nodal metastases was 33 months (95% CI: 31–NA), while in the subset with nodal metastases only was 30 months (95% CI: 13–NA; *p* = 0.72).

### Toxicity

Treatment was feasible and well tolerated. All patients reported at least one grade (G) 1–2 AE related to enzalutamide per the clinician’s judgment, with the most common being fatigue (49%) and hypertension (33%). Six G3 AEs were reported leading to the permanent discontinuation of treatment: two cases of hypertension, one seizure, one fatigue, one diarrhea, and one headache. No G 4–5 AEs were reported. Regarding the patient who experienced a seizure, it should be noted that the patient had an unknown medical history of previous epileptic seizures that were not reported at the time of enrollment.

### Exploratory endpoint

In all, 55 patients underwent liquid biopsy ARv7 detection, as blood samples were not collected for 12 of 67 patients included in the final analysis. Among the 55 patients, the ARv7 assessment failed in one case as the collected blood sample reached the central laboratory 48 h after the blood draw. Detectable levels of ARv7 were observed in 13/54 (24%) patients, with patients having liver metastases showing undetectable levels of ARv7. In 48 cases, the 3-mo-DCR was matched with ARv7 detection by liquid biopsy using blood samples collected at the start of the treatment. Patients with detectable liquid biopsy ARv7 levels were significantly associated with poor treatment response (two-tailed value: *p* = 0.034, chi-square test) compared to those with undetectable ARv7 at baseline. Moreover, the percentage of patients with detectable ARv7 in their liquid biopsies decreased from 57% to 25% and 15% and was null in patients classified as PD, SD, PR, and CR, respectively, thus mirroring the degree of response, although only a trend toward significance was observed (*p* = 0.15).

Time-dependent analyses were conducted to explore the impact of baseline ARv7 status on PFS and OS. Patients defined as ARv7 positive at baseline had a significantly shorter PFS (*p* = 0.005) and OS (*p* = 0.0055).

## Discussion

Subgroup analysis of pivotal trials supports the benefit of first- or second-line hormonal therapy in men with mCRPC and visceral metastases.^11–13^ However, no prospective trials have been specifically designed to test this treatment in this specific population. The COU-AA-302 trial^7^ of abiraterone acetate in chemotherapy-naïve men with mCRPC completely excluded patients with visceral disease. On the other hand, the study of enzalutamide in mCRPC patients included patients with visceral disease in both pre- and post-docetaxel settings.^12,13^ Thus, at the time of designing this study, we considered enzalutamide as a reasonable treatment option for men with mCRPC who had measurable lesions including visceral metastases. In this paper, we report the results from the Excalibur trial, the first prospective study specifically designed to test the efficacy and safety of the hormonal agent enzalutamide in men with mCRPC and one measurable metastatic lesion according to RECIST 1.1 including visceral disease and/or extra-regional lymph nodes. Excalibur successfully reached its primary endpoint achieving a significant 3-mo-DCR of 72% while demonstrating a manageable safety profile. Moreover, the detection of the ARv7 variant in liquid biopsy was associated with 3-mo-DCR and mirrored the RECIST response. As known, up to 30% of patients with CRPC present with visceral metastases, a long-recognized poor prognosis factor. Our findings support the recognition of the prognostic value of visceral disease while suggesting that enzalutamide has efficacy for mCRPC patients with more advanced diseases associated with visceral involvement. Notably, a remarkable 3-mo-DCR rate was obtained in patients with lung and/or liver involvement, not exclusive to patients with nodal involvement. However, the prognosis of patients with lung-only disease is significantly better than those with liver metastases. Only 22% of patients received previous treatment with docetaxel in the mCRPC phase; thus, the majority of patients included in the Excalibur study are represented by a pre-docetaxel subset, suggesting the efficacy of enzalutamide regardless of the line of therapy. Meanwhile, the treatment paradigm for mHSPC has radically changed in recent years, with new hormonal agents such as abiraterone, apalutamide, and enzalutamide demonstrating prolonged survival with proven efficacy even in the case of visceral disease.^14,15,25–28^ Considering all the limitations and the necessary caution in the interpretation of indirect comparisons, survival outcomes appeared to be similar to those previously reported in the COU-AA-301,^11^ AFFIRM,^12^ and PREVAIL^13^ trials. PFS and OS were 17 months (95% CI: 13–25) and 33 months (95% CI: 30–NA) in the Excalibur trial *versus* 5.6 and 12.9 in the visceral subset of COU-AA-301 trial^11^ *versus* 2.9 and 9 in the liver subset of AFFIRM trial^12^ and 13.9 and 17 in the lung subset of AFFIRM trial^12^ *versus* 5.3 and 18.9 months in the liver subset of the PREVAIL^13^ trial and NR and 32.4 months in the lung subset of PREVAIL trial.^13^ However, caution is necessary when interpreting those results since the Excalibur trial was not designed to properly evaluate PFS and/or OS, preventing a definitive conclusion. Moreover, the limited number of patients in this study makes definitive conclusions difficult to draw. Regarding safety, enzalutamide was well-tolerated in patients with visceral disease, with the tolerability profile consistent with previously reported results and no new safety signals. Liquid biopsy in prostate cancer is a groundbreaking diagnostic tool that revolutionizes the way we detect and monitor this disease. Unlike traditional biopsies that require invasive procedures, liquid biopsy involves analyzing a patient’s blood sample to identify circulating tumor DNA (ctDNA), CTCs, or other biomarkers.^29^ This non-invasive approach offers several advantages, including the ability to capture a comprehensive picture of tumor alterations and monitor treatment response over time. It holds great promise in personalizing treatment strategies, enabling timely interventions, and improving outcomes for individuals with prostate cancer.

Following the seminal study by Antonarakis *et al.*,^22^ ARv7 detection in liquid biopsies gained momentum^30^ and holds promise for becoming a clinically useful tool. Despite most studies on ARv7 supporting its prognostic and predictive relevance, a direct comparison among studies is hindered by the use of different biological materials (whole blood, peripheral blood mononuclear cells (PBMC), CTCs, exosomes, plasma) and different detection approaches (qRT-PCR, ddPCR, IHC, and RNAseq). In our study, we employed a positive CTC-enrichment approach with a commercially available kit followed by qRT-PCR, and we obtained ARv7 detection levels falling in the range described in the literature (20–60%). Notably, even in the setting of visceral disease, the detection of ARv7 in liquid biopsies collected at the start of treatment showed a significant association with the lack of clinical benefit. In fact, ARv7 positivity rates reached 57% in the group of patients experiencing progression, compared to 50% and 35% positivity rates observed in previous studies when considering PSA or radiological response, respectively. However, small patient numbers and slight technical modifications may account for these differences. This study presents a series of limitations. First, due to the multicentric nature of the study, ARv7 could not be analyzed in all participating centers, and most samples refer to patients enrolled in the study-coordination center (INT) where the liquid biopsy assays were centralized. Second, the study has not been properly designed for prognostic assessment. Third, due to the small number of patients undergoing ARv7 assessment, a subgroup analysis by type of visceral metastasis was not feasible. Nonetheless, the results strongly favor the use of liquid biopsy for ARv7 detection, even in this specific setting of CRPC patients. Moreover, neuroendocrine differentiation has been associated with a poor prognosis.^2^ Unfortunately, our study will not provide information on neuroendocrine differentiation as sample collection was not planned.

## Conclusion

The Excalibur trial successfully achieved its primary endpoint, demonstrating that enzalutamide is an effective and safe therapeutic option for mCRPC patients with more advanced diseases associated with visceral involvement. In the explorative part of the study, ARv7 detection was found to be significantly associated with the clinical benefits observed in this specific patient subgroup.

## Declarations

**Ethics approval and consent to participate:** The study was approved by the local ethics committees of all participating institutions and was executed in accordance with Good Clinical Practice and the Declaration of Helsinki [ICH GCP E6(R2)]. All patients provided written informed consent before study procedures.

**Consent for publication:** Not applicable.

**Availability of data and materials:** Data are available upon reasonable request.
